# Editorial: Kidney and Distant Organ Crosstalk in Health and Disease

**DOI:** 10.3389/fphys.2021.712535

**Published:** 2021-07-01

**Authors:** Natalia López-Andrés, Frederic Jaisser, Jonatan Barrera-Chimal

**Affiliations:** ^1^Cardiovascular Translational Research, Complejo Hospitalario de Navarra (CHN), Universidad Pública de Navarra (UPNA), IDISNA, Navarrabiomed (Miguel Servet Foundation), Pamplona, Spain; ^2^INSERM, UMRS 1138, Centre de Recherche des Cordeliers, Sorbonne Université, Université de Paris, Paris, France; ^3^Université de Lorraine, INSERM Centre d'Investigations Cliniques-Plurithématique 1433, UMR 1116, CHRU de Nancy, French-Clinical Research Infrastructure Network (F-CRIN) INI-CRCT, Nancy, France; ^4^Instituto de Investigaciones Biomédicas, Universidad Nacional Autónoma de México, Ciudad Universitaria, Mexico City, Mexico; ^5^Laboratorio de Fisiología Cardiovascular y Trasplante Renal, Unidad de Investigación UNAM-INC, Instituto Nacional de Cardiología Ignacio Chávez, Mexico City, Mexico

**Keywords:** cardiorenal, kidney disease, hemodialysis, inflammation, fibrosis

Kidney disease is a health condition affecting a high number of patients with an estimated worldwide prevalence of 10% (Jager et al., 2019). Kidney dysfunction and the resulting accumulation of uremic toxins and chronic inflammation contribute to secondary injury in organs like the heart, vessels, lungs, gut, brain, liver, among others (Malek, 2018; Shang et al., 2020; Ambruso et al., 2021; Lai et al., 2021). On the other hand, kidney disease might be the result of co-morbidities or due to a primary insult in another organ (Ronco et al., 2018). The mechanisms by which this kidney-distant organ communication occurs remain partially understood. This Research Topic compiled original investigation and review papers that explored the connection between the kidneys and other organs/co-morbidities and result in the proposal of novel biomarkers, therapeutic strategies and bring new insights into the molecular mechanisms linking kidney disease with other organs.

## The Kidneys and the Cardiovascular System

The close relationship between the kidneys and the cardiovascular system has long been recognized. While the main cause of death in chronic kidney disease (CKD) patients is related to major cardiovascular events, acute or chronic heart failure may also result in kidney damage (Ronco et al., 2018; Ricci et al., 2021). In this sense, a common complication in cardiac surgery patients is acute kidney injury (AKI) (O'Neal et al., 2016), however its diagnosis is limited due to the lack of sensitive and early biomarkers. To address this issue, Chen et al. explored the levels of 48 cytokines in the plasma from patients undergoing cardiac surgery. Twenty-four hours after cardiac surgery, 13 cytokines showed a remarkable increase in patients that developed AKI as compared to patients without AKI. Importantly, interferon-γ and stem cell growth factor-β plasma levels efficiently discriminated between the severity levels of AKI. Testing these biomarkers in larger cohorts will bring new insights into their usefulness to establish an early diagnosis of AKI in cardiac surgery patients. Continuing with the close connection between the cardiovascular system and kidney disease, Lim T. et al. performed a single-center retrospective cohort study in which they found that in type 2 diabetic patients, increased peripheral arterial stiffness determined by brachial-ankle pulse wave velocity tests may be risk factor for renal disease progression (HR: 8.480, *p* = 0.014).

Three review papers addressed the mechanisms linking CKD and cardiovascular complications. Lim K. et al. focused on integrative physiology changes that occur in the CKD patient and that involve multisystemic responses resulting in perturbations of the oxygen transport system, and highlighted the possible application of cardiopulmonary exercise testing to evaluate cardiovascular functional alterations in patients with kidney disease. Junho et al. reviewed the evidence around the role that calcium/calmodulin-dependent protein kinases (CaMKs) play in the cardiorenal syndrome pathology through modulating inflammation and oxidative stress. Though more studies are required, the current evidence points out for a role of CaMKs in CKD-associated cardiac injury. Finally, Lowenstein and Nigam, present an integrative view of the multi-organ effects of uremic toxin accumulation when kidney function is compromised with special focus on the role of uremic toxins and drug transporters in inter-organ communication (intestine-liver-kidney-brain) and inter organismal communication, for example the host with the microbiome.

## Novel Therapeutic Options for Kidney Disease

A major complication of diabetes is diabetic kidney disease, for which the therapeutic options are limited (Barrera-Chimal and Jaisser, 2020). Xiao et al. studied the effect of the treatment with oxymatrine (OMT) in renal fibrosis induced by type 2 diabetes in mice. Following 8 weeks of treatment with OMT, diabetic mice presented decreased renal expression of fibrotic markers and increased expression of E-cadherin as an epithelial marker. This protective effect was mediated via upregulation of the inhibitor of differentiation-2 which would bind and inhibit Twist-mediated epithelial to mesenchymal transition. This mechanism was corroborated in tubular epithelial cells cultured in high glucose. Another strategy to target renal fibrosis was tested in the article by Rui-Zhi et al. who showed that the *Astragalus mongholicus* Bunge and Panax notoginseng formula and Bifidobacterium administration has functional and structural renal protective effects in a mouse model of CKD induced by 5/6th nephrectomy through the inhibition of inflammatory M1 macrophages via Mincle/NfkB pathway inhibition in the kidney. Similarly, mincle/NFkB pathway was also inhibited in the intestine, leading to the restoration of the intestinal barrier in CKD mice and a recovery of the intestinal flora similar to the one present in normal mice. Thus, by inhibiting inflammation in both, the kidney and the gut this treatment reduced renal and intestine injury induced by CKD, highlighting the role of inflammation in intestine changes observed during CKD.

Kidney injury is often associated to an imbalance of immune cell activation leading to uncontrolled and chronic inflammatory processes affecting the renal structure and function (Meng et al., 2014). Cell based therapy with T regulatory cells (Treg) as a therapy in kidney disease was studied by Lu J. et al. First, the authors showed that in RNA-seq datasets from mouse and human diseased kidneys, immune system activation related genes are upregulated with a specific enrichment of genes related to Tregs. Increased Treg abundance was confirmed in a mouse model of Adriamycin induced kidney injury. Furthermore, adoptive spleen Treg transfer from healthy to Adriamycin treated mice protected against renal structural injury. Co-culture of Treg with M2c anti-inflammatory macrophages increased Treg expression of chemotactic molecules which could facilitate its recruitment to the inflamed kidney site to resolve inflammation. This data underlines the importance of regulated inflammation in resolving kidney injury.

## Complications Associated to Advanced CKD Patients in Dialysis

The understanding and characterization of the complications that develop in advanced CKD patients undergoing dialysis is important in order to adequately monitor them and provide a good care to improve survival and life quality of these patients.

Hematological alterations other than anemia are a less explored complication observed in hemodialysis (HD) patients for which the risk factors need to be known by healthcare providers to deliver an accurate management. In a single-center study, Lee et al. explored the risk factors associated to leukopenia or thrombocytopenia in HD patients. Older age (>-60) at dialysis initiation was found to be a predictor for chronic leukopenia and thrombocytopenia, while chronic liver disease and high serum ferritin levels (>800 mg/dL) were risk factors for chronic thrombocytopenia. In patients in maintenance HD, Zhang Y. et al. showed that moderate to severe tricuspid regurgitation occurs in 62.6% of the patients and that sodium, decreased fractional shortening and increased right atrium diameter were independently associated to moderate to severe tricuspid regurgitation. Zhang Q. et al. showed that increased arterial stiffness determined by pulse wave velocity together with high plasma galectin-3 levels predicted mortality and cerebral and cardiovascular events in stable HD patients. These studies are important as proof of concept that these parameters might be used to predict adverse cardiovascular events in CKD patients and further confirmation is required in larger cohorts.

Lu H. et al. present an observational retrospective single-center study to assess the prevalence of hyponatremia in hospitalized patients and its related adverse outcomes. The authors found a prevalence of hyponatremia of 32.5% and was associated with higher hospital costs and length of stay and in-hospital and 30-day mortality.

## Kidney Disease and Neurological Disorders

Kidney disease is often associated to neurological disorders. While the mechanisms remain elusive, ischemic AKI has been associated with a long-term risk of stroke and dementia (Tanaka and Okusa, 2020). In the investigation by Burek et al. the authors created an *in vitro* model to gain insights into the kidney-brain crosstalk during disease. They showed that hypoxic and glucose starved tubular cells co-cultured with brain microvascular endothelial cells (BMEC) on a transwell system and with the addition of uremic toxins induced changes in the expression of blood brain barrier (BBB) transporters, receptors and tight junction proteins in BMEC. This changes in BBB efflux and influx transporter expression were validated in brain capillaries isolated from mice after 24 h of ischemic AKI induction.

## Effect of COVID-19 on Kidney Health

Over the past 18 months, COVID-19 pandemic has affected several millions worldwide. Numerous studies have recognized the kidney as a target for the severe acute respiratory syndrome coronavirus 2 (SARS-CoV-2) (Vijayan and Humphreys, 2020). In a timely analysis, Hong et al. presented the renal function parameters observed in a small cohort of patients diagnosed with COVID-19 at the beginning of the pandemics. Moreover, Zhou et al. performed a meta-analysis including 52 studies to evaluate the effect of COVID-19 on CKD or AKI patients. The prevalence of CKD or AKI was higher in those patients developing severe COVID-19 and in deceased cases vs. survivors. Worse prognosis of COVID-19 is observed in kidney patients, thus, focusing attention on patients with preexisting kidney disease or new kidney injury as a consequence of COVID-19 may help to prevent complications and mortality.

## Conclusions

The contributions to this Research Topic illustrate the complexity of the communication between the kidneys and other organs in health and disease conditions, providing new therapeutic and diagnosis tools, and contributing to mechanistic insights into the connection of kidney disease and distant organ disorders. The review articles also highlight that further efforts are needed in order to gain complete understanding of the molecular processes involved in these pathological connections, which will help to propose novel therapeutic avenues for kidney disease and its associated distant organ injuries.

## Author Contributions

All authors contributed to the editorial, approved the submitted version, and performed editorial assignments for this Research Topic.

## Conflict of Interest

The authors declare that the research was conducted in the absence of any commercial or financial relationships that could be construed as a potential conflict of interest.

## References

[B1] Ambruso S. L. Gil H. W. Fox B. Park B. Altmann C. Bagchi R. A.. (2021). Lung metabolomics after ischemic acute kidney injury reveals increased oxidative stress, altered energy production, and ATP depletion. Am. J. Physiol. Lung Cell Mol. Physiol. 10.1152/ajplung.00042.2020 33949208 PMC8321856

[B2] Barrera-Chimal J. Jaisser F. (2020). Pathophysiologic mechanisms in diabetic kidney disease: a focus on current and future therapeutic targets. Diabetes Obes. Metab. 22 (Suppl. 1), 16–31. 10.1111/dom.13969 32267077

[B3] Jager K. J. Kovesdy C. Langham R. Rosenberg M. Jha V. Zoccali C. (2019). A single number for advocacy and communication-worldwide more than 850 million individuals have kidney diseases. Kidney Int. 96, 1048–1050. 10.1016/j.kint.2019.07.012 31582227

[B4] Lai H. J. Zhan Y. Q. Qiu Y. X. Ling Y. H. Zhang X. Y. Chang Z. N.. (2021). HMGB1 signaling-regulated endoplasmic reticulum stress mediates intestinal ischemia/reperfusion-induced acute renal damage. Surgery. 170, P239–P248. 10.1016/j.surg.2021.01.042 33745733

[B5] Malek M. (2018). Brain consequences of acute kidney injury: focusing on the hippocampus. Kidney Res. Clin. Pract. 37, 315–322. 10.23876/j.krcp.18.0056 30619687 PMC6312775

[B6] Meng X. M. Nikolic-Paterson D. J. Lan H. Y. (2014). Inflammatory processes in renal fibrosis. Nat. Rev. Nephrol. 10, 493–503. 10.1038/nrneph.2014.114 24981817

[B7] O'Neal J. B. Shaw A. D. Billings F. T. T. (2016). Acute kidney injury following cardiac surgery: current understanding and future directions. Crit. Care 20:187. 10.1186/s13054-016-1352-z 27373799 PMC4931708

[B8] Ricci Z. Romagnoli S. Ronco C. (2021). Cardiorenal syndrome. Crit. Care Clin. 37, 335–347. 10.1016/j.ccc.2020.11.003 33752859

[B9] Ronco C. Bellasi A. Di Lullo L. (2018). Cardiorenal syndrome: an overview. Adv. Chronic Kidney Dis. 25, 382–390. 10.1053/j.ackd.2018.08.004 30309455

[B10] Shang Y. Madduma Hewage S. Wijerathne C. U. B. Siow Y. L. Isaak C. K. and O. K. (2020). Kidney ischemia-reperfusion elicits acute liver injury and inflammatory response. Front. Med. 7:201. 10.3389/fmed.2020.00201 32582723 PMC7280447

[B11] Tanaka S. Okusa M. D. (2020). Crosstalk between the nervous system and the kidney. Kidney Int. 97, 466–476. 10.1016/j.kint.2019.10.032 32001065 PMC7039752

[B12] Vijayan A. Humphreys B. D. (2020). SARS-CoV-2 in the kidney: bystander or culprit? Nat. Rev. Nephrol. 16, 703–704. 10.1038/s41581-020-00354-7 32929200 PMC7487445

